# Prevalence of dyslipidemia and predictive value of anthropometric indicators among children and adolescents in the Tibetan Plateau

**DOI:** 10.3389/fnut.2025.1531197

**Published:** 2025-03-28

**Authors:** Ming-jian Nie, Rui-zhe Sun, Chao-qun Fan, Xi Fei, Hong-juan Li

**Affiliations:** ^1^School of Sport Science and Key Laboratory of the Ministry of Education of Exercise and Physical Fitness, Beijing Sport University, Beijing, China; ^2^Tibet Institute of Sport Science, Lhasa, China; ^3^Key Laboratory of Plateau Sports and Health of Xizang Autonomous Region, Lhasa, China; ^4^National Research Centre for Physical Fitness and Scientific Fitness, China Institute of Sport Science, Beijing, China; ^5^Department of Social Sports, Beijing College of Sports, Beijing, China

**Keywords:** dyslipidemia, anthropometry, Tibet, plateau, children, adolescent

## Abstract

**Background/Objectives:**

Dyslipidemia during adolescence has been associated with in-creased risk of cardiovascular disease throughout life; however, its prevalence and anthropometric predictors remain understudied in high-altitude populations. This study aimed to investigate the prevalence of dyslipidemia among children and adolescents in the Tibetan Plateau and evaluate the predictive capability of 15 anthropometric indices [mid-upper arm circumference (MUAC), subscapular skinfold thickness (SST), body mass index (BMI), relative fat mass (RFM), mid-upper arm-to-height ratio (MaHtR), etc.] for dyslipidemia in this population.

**Methods:**

A total of 367 Tibetan and Han Chinese students aged 8–17 years were recruited from six schools in Chengguan District, Lhasa (altitude 3,650 m). Anthropometric measurements and lipid profiles were assessed. Dyslipidemia was primarily diagnosed according to the Expert Consensus on Prevention and Treatment of Dyslipidemia in Chinese Children and Adolescents. Receiver Operating Characteristic (ROC) analysis was employed to examine the predictive ability of anthropometric indices for dyslipidemia.

**Results:**

One in four students (24.25%) had dyslipidemia, with low high-density lipoprotein cholesterol (HDL-C) being found as the predominant phenotype (17.98%). Han Chinese students exhibited higher dyslipidemia prevalence compared to their Tibetan counterparts (13.62 vs. 10.63%), with higher rates observed among females than males (14.17 vs. 10.08%). Junior high school students demonstrated the highest prevalence among the three grade groups (11.99 vs. 6.54 vs. 5.72%). Most anthropometric indices showed the area under the ROC (AUC) values below 0.6, with only MUAC, SST, BMI, RFM, and MaHtR demonstrating significant diagnostic accuracy. Their highest AUC values in subgroups reached only 0.651 and were not consistently applicable across all subpopulations.

**Conclusions:**

Children and adolescents in the Tibetan Plateau demonstrate a high prevalence of dyslipidemia, particularly among Han Chinese students, girls and junior high school students. Low HDL-C emerged as the primary dyslipidemia phenotype. Most anthropometric indices demonstrated limited diagnostic capability for dyslipidemia among plateau children and adolescents, with only BMI, SST, MUAC, MaHtR, and RFM showing weak yet promising diagnostic value.

## 1 Introduction

Dyslipidemia is one of the most common chronic conditions, characterized by elevated levels of total cholesterol (TC), triglycerides (TG), and low-density lipoprotein cholesterol (LDL-C), and/or decreased levels of high-density lipoprotein cholesterol (HDL-C). Dyslipidemia is a leading cause of morbidity and mortality in most countries (1) and is a significant risk factor for cardiovascular diseases (2), diabetes (3), and stroke (4). While most types of dyslipidemia are not associated with severe adverse health outcomes during childhood, growing evidence has suggested that abnormal lipid levels during adolescence increase the risk of cardiovascular diseases throughout the lifespan (5). Therefore, this developmental stage presents a critical window of opportunity to implement effective measures to prevent cardiovascular diseases and other clinical outcomes in adulthood. Screening and controlling dyslipidemia from childhood is a feasible and necessary health strategy.

The Tibetan Plateau has an average elevation exceeding 4,000 meters, making it one of the highest human habitats in the world. The plateau environment is characterized by low temperature, low oxygen, strong ultraviolet radiation, and large climate differences, has a profound impact on the human body (6, 7), and lipid metabolism differs from that in lowland areas (8, 9). Tibetans and Han Chinese are the main residents of the Tibetan Plateau, accounting for 90.48 and 8.17% of the total population in the region, respectively (10). Tibetans are the indigenous people of the Tibetan Plateau, while Han Chinese are mainly migrants from lowland areas. The two ethnic groups have very different daily living habits and dietary structure. The traditional Tibetan diet is dominated by beef, mutton, dairy products, and butter tea, lacking vegetables and fruits, and high fat intake tends to increase with altitude (11). This unique dietary structure, combined with the hypoxic environment, easily leads to dyslipidemia, which has become one of the major diseases threatening the health of the Tibetan population (12). One study reported that the prevalence of dyslipidemia among Tibetan adults in Changdu reached 42.7% (13), far higher than the national average of 34.0% in the same period (14). Previous studies have also found that lipid levels in Tibetan adults exhibit a U-shaped change with increasing altitude (11). Studies in the Andes Plateau have shown that children living at high altitudes have a higher prevalence of hypertriglyceridemia and low HDL-C levels (15), and total cholesterol levels are also significantly higher than those in children at low altitudes (16). However, as a special geographical and demographic unit, the current status of blood lipid levels in children and adolescents on the Tibet Plateau has not been adequately studied, and there is currently no systematic report on the detection rate of dyslipidemia in this region. Given the potential impact of altitude on lipid metabolism and the unique characteristics of the local population, understanding the prevalence and predictors of dyslipidemia in this region is crucial for developing targeted prevention and intervention strategies.

In clinical practice, the detection of dyslipidemia typically requires blood sampling and laboratory testing. Although the cost of measuring an individual's lipid profile is limited, the economic burden imposed by the large population size can be enormous for a country. In China, the annual cost of dyslipidemia screening is as high as $16 billion (17). Furthermore, incorporating such invasive testing into routine health monitoring for adolescents on a large scale is complex (18), and may face many challenges in resource-limited high-altitude regions. Therefore, identifying simple, non-invasive, and economical alternative indicators to predict dyslipidemia is of substantial practical importance. It is generally believed that excessive body fat is closely related to blood lipids (19). Based on this assumption, researchers have evaluated a series of anthropometric indices used to measure body shape or fat distribution to explore their applicability in assessing lipid levels and screening for dyslipidemia (20). Traditional anthropometric indices, such as body mass index (BMI), waist circumference (WC), waist-to-height ratio (WHtR), skinfold thickness, and their derived indices, have been widely studied and proven to be associated with lipid levels or dyslipidemia in different populations (21–25). Simultaneously, some emerging anthropometric indices in recent years, such as mid-upper arm circumference [MUAC; (26)], a body shape index [ABSI; (27, 28)], relative fat mass [RFM; (29, 30)], and body roundness index [BRI; (31)], have also been found to diagnose obesity (32), detect atherosclerosis (33), or estimate an individual's body fat percentage (29, 31) more accurately in certain populations, and have similarly shown the ability to detect dyslipidemia (17, 20, 21, 34, 35). However, despite the good performance of these indices in some studies, their validity and applicability remain controversial (20, 36–38). The applicability of these indices in different populations has not been fully validated, especially in the pediatric population and high-altitude environments. Previous studies have shown that in low-altitude areas, some anthropometric indices can well predict dyslipidemia (17, 20, 22). However, whether this predictive ability still exists in the plateau environment and which anthropometric indices have the highest diagnostic value for dyslipidemia in Tibetan plateau children and adolescents are questions that currently lack clear answers. Therefore, specifically evaluating the diagnostic utility of these anthropometric indices in children and adolescents on the Qinghai-Tibet Plateau can clarify their applicability in a unique physiological environment and provide information for screening strategies in specific regions.

Approximately 63 million people worldwide live at altitudes above 2,500 meters, and more than 17 million live above 3,500 meters (39). However, there is limited research on the prevalence of dyslipidemia and the diagnostic value of anthropometric indices among children and adolescents in high-altitude regions. Based on these considerations, this study aimed to investigate the prevalence of dyslipidemia among children and adolescents in the Tibetan Plateau, and to comprehensively evaluate the ability of anthropometric indices to predict dyslipidemia in this population.

## 2 Materials and methods

### 2.1 Design and participants

The data for this cross-sectional study were obtained from the “Investigation on Physical Fitness and Health Behaviors of Tibetan School-Age Children and Adolescents (IPFHB-TSCA)” (6, 40). We randomly selected six schools (two each from primary, junior high, and senior high schools) in Lhasa's Chengguan District. From each grade level, 48 students were enrolled with an equal distribution of sex and ethnicity. A total of 432 students aged 8–17 years (grades 4–12) were recruited. The selection of Chengguan District as the sampling area was based on the fact that over half of the senior high schools and two-thirds of the junior high schools are located in the Chengguan District of Lhasa City.

Eligible participants were Tibetan or Han Chinese students aged 8–17 years residing in the study area, capable of engaging in routine physical activities, and able to comprehend and comply with measurement protocols. Exclusion criteria included individuals with severe physiological disabilities, a history of specialized sports training, vigorous exercise the day before the test, or self-reported acute/chronic diseases that could potentially affect the detection of blood lipid levels (e.g., colds, metabolic diseases, liver diseases, kidney diseases, or thyroid dysfunction). Before the start of the study, written informed consent was provided by the students and their guardians. Participants were informed that they could withdraw at any time, and all students' names were coded to ensure confidentiality.

### 2.2 Measure

#### 2.2.1 Demographic characteristics

Demographic information, including sex, ethnicity, date of birth, and grade was obtained through a questionnaire. The main framework of the questionnaire is based on the localization revision of the WHO's Health Behavior in School-Aged Children (HBSC) study (41). The questionnaires could be used well in Lhasa primary and secondary schools according to the pilot study. The participants were primarily divided into two ethnic groups: Tibetan (indigenous population) and Han (immigrants or descendants of immigrants from lowland areas), reflecting different lifestyles and genetic backgrounds. Furthermore, participants were categorized into three stages based on their grade level: primary school (PS)—grades 4 to 6, junior high school (JHS)—grades 7 to 9, and senior high school (SHS)—grades 10 to 12.

#### 2.2.2 Anthropometric measurements and derived indicator calculations

Anthropometric data were collected by trained investigators following standardized protocols. Height and body weight were measured separately using a validated electronic stadiometer (GMCS-IV GJ-II) and electronic scale (GMCS-IV RCS-II), both manufactured by Jianmin (Beijing Xindong Huateng sports Facilities Co., Ltd, Beijing, China). For height measurement, participants were instructed to stand barefoot in an upright position with continuous contact between their back, buttocks, heels and the vertical rod of the stadiometer. The Frankfurt horizontal plane was used for head alignment, and measurements were recorded to the nearest 0.1 cm. For weight assessment, participants removed footwear and heavy outer garments, standing motionless on the calibrated electronic scale. Weight values were documented to the nearest 0.1 kg.

WC and MUAC were measured using a flexible non-elastic anthropometric tape (SECA 201, Seca GmbH and Co. KG, Germany; manufactured under license in China) and all measurements were recorded to the nearest 0.1 cm, with the WC measurement taken at the midpoint between the lower rib margin and the iliac crest and the MUAC measurement taken at the midpoint of the acromion-radiale line.

Triceps skinfold thickness (TST), subscapular skinfold thickness (SST), and abdominal skinfold thickness (AST) were measured using a skinfold caliper (GMCS-PZQ, JianMin model, Beijing Xindong Huateng Sports Facilities Co., Ltd., China), recorded to the nearest 0.1 mm, with measurements taken at the midpoint of the posterior upper arm (TST), ~2 cm below the inferior angle of the scapula (SST), and 1 cm to the right of the umbilicus (AST). The final values represent the average of two measurements. For waist circumference, mid-upper arm circumference, and skinfold thickness measurements, participants were asked to expose the measurement sites to ensure direct skin contact and accuracy.

Using the aforementioned measurements, the following anthropometric indices were calculated using their respective formulas: BMI, WHtR, mid-upper arm-to-height ratio (MaHtR), conicity index [C-index; (42)], BRI (31), ABSI (27, 28), RFM (29, 30), sum of skinfold thickness (SuST), and body fat percentage [BF%, calculated using skinfold thickness equations; (43)].


$$\begin{array}{r} BMI=Weight(kg)/{\left[Height(m)\;\right]}^{2}\\ WHtR=WC(cm)/Height(cm)\\ MaHtR=\;MUAC(cm)/Height(cm)\\ C-index=0.109^{-1}WC\left(m\right){\left[\frac{Weight\left(kg\right)}{Height\left(m\right)}\right]}^{-\frac{1}{2}}\\ BRI=364.2-365.5\times \sqrt{1-\frac{{\left(\frac{WC}{2\pi }\right)}^{2}}{{\left(0.5Height\right)}^{2}}} \end{array}$$

ABSI was calculated using two versions of the formula: the original version (ABSI-OR) developed by Krakauer and Krakauer (27), and the version developed by Xu et al. (28) specifically for Chinese adolescents (ABSI-CN):


$$\begin{array}{r} ABSI-OR=\frac{WC\left(m\right)}{BMI^{2/3}\times Height(m{)}^{1/2}}\\ ABSI-CN=\frac{WC\left(m\right)}{BMI^{0.45}\times Height(m{)}^{0.55}} \end{array}$$

RFM was calculated as follows (29, 30), where sex is 0 for boys and 1 for girls:


$$\begin{array}{r} RFM\left(8\sim 14years\right)=74-22\times \left[\frac{Height\left(m\right)}{WC\left(m\right)}\right]+12\times sex\\ RFM\left(15\sim 19years\right)=64-20\times \left[\frac{Height\left(m\right)}{WC\left(m\right)}\right]+12\times sex \end{array}$$

SuST was calculated as follows:


SuST=TST+SST+AST

Additionally, BF% were calculated using the skinfold thickness equations proposed by Slaughter et al. [BF-S%; (43)]. For subjects with sum triceps and subscapular skinfolds >35 mm, BF-S% was calculated as follows:


$$\begin{array}{l} BF-S\left(girl\right)\%=0.546\left(TST+SST\right)+9.7\\ BF-S\left(boy\right)\%=0.783\left(TST+SST\right)+1.6 \end{array}$$

For subjects with sum triceps and subscapular skinfolds < 35 mm, BF-S% was calculated as follows:


$$\begin{array}{l} BF-S\left(girl\right)\%=1.33\left(TST+SST\right)-0.013{\left(TST+SST\right)}^{2}-2.5\\ BF-S\left(boy\right)\%=1.21\left(TST+SST\right)-0.008{\left(TST+SST\right)}^{2}-3.4 \end{array}$$

#### 2.2.3 Blood lipid profiles and definition of dyslipidemia

Fasting blood samples were collected from the ulnar vein by trained personnel after a 12-h overnight fast. Biochemical analysis of the lipid profile, including TC, HDL-C, LDL-C, and TG, was performed using standardized methods under strict quality control at a certified laboratory (KingMed Diagnostics Center, Chengdu, China).

Dyslipidemia was primarily determined according to the expert consensus on the prevention and treatment of dyslipidemia among Chinese children and adolescents [ECC criteria; (44)]. In detail, subjects were classified as having dyslipidaemia if they met one or more of the following criteria regarding abnormal lipid level: TC ≥ 5.18 mmol/L, LDL-C ≥ 3.37 mmol/L, HDL-C ≤ 1.04 mmol/L, TG ≥ 1.70 mmol/L.

### 2.3 Statistical analysis

Statistical analyses were performed using JMP Pro (version 17.0) and IBM SPSS 27 software. The Shapiro-Wilk test was used to verify if all parameters followed a normal distribution. Non-normally distributed variables were described using median and interquartile range (Q1–Q3). Categorical variables were presented as frequencies and per-centages. For comparisons between continuous variables, independent samples *t*-test (for normal data) or Wilcoxon rank-sum test (for non-normal data) were used for two-group comparisons, while ANOVA (for normal data with homogeneous variances) or the Steel-Dwass test (for non-normal data or unequal variances) were used for multi-group comparisons. For comparisons between groups of categorical variables, Pearson's chi-square test was used. For comparisons between three groups of categorical variables, the Bonferroni correction was applied to the Pearson chi-square test significance level to determine the results of pairwise comparisons. Receiver operating characteristic (ROC) analysis was performed to calculate the area under the ROC curve (AUC) to evaluate the predictive ability of anthropometric indices for dyslipidemia. DeLong's test was used to compare the performance difference between two ROC curves. A *p* < 0.05 was considered statistically significant, but for pairwise comparisons among multiple groups, the significance level was adjusted to 0.0167 (0.05 ÷ 3) using the Bonferroni method.

## 3 Results

### 3.1 Participants' sociodemographic characteristics

Of the 432 participants, 401 had both lipid profiles and anthropometric measurements available, 34 participants aged ≥18 years were excluded. A total of 367 children and adolescents were included in our analysis. The demographic characteristics of the study participants are shown in Table 1.

**Table 1:** Characteristics of the study population.

**Variables**	**Ethnicity**			**Sex**			**Total**
	**Tibetan**	**Han Chinese**	***p*** **-value**	**Male**	**Female**	***p*** **-value**	
N	182 (49.59%)	185 (50.41%)	1.0000	188 (51.23%)	179 (48.77%)	1.0000	367 (100.00%)
Age (years)	13.10 (11.38, 15.43)	13.40 (11.25, 15.55)	0.7528	13.30 (11.30, 15.95)	13.20 (11.30, 15.20)	0.3234	13.30 (11.30, 15.50)
Height (cm)	156.05 (143.77, 162.65)	152.89 (141.40, 163.30)	0.4104	158.40 (142.33, 168.10)	152.69 (142.30, 158.50)	< 0.0001	154.40 (142.30, 162.80)
Weight (kg)	45.95 (32.98, 53.38)	42.30 (32.25, 52.05)	0.1557	47.30 (32.93, 54.50)	42.60 (32.20, 50.40)	0.0272	45.20 (32.70, 52.80)
WC (cm)	65.50 (59.50, 69.65)	63.50 (57.00, 67.55)	0.0201	65.25 (58.28, 70.00)	62.50 (57.80, 68.00)	0.0126	64.30 (58.00, 68.90)
MUAC (cm)	22.00 (19.28, 24.00)	21.50 (18.90, 24.00)	0.6079	22.00 (18.80, 24.00)	21.70 (19.20, 23.50)	0.3997	22.00 (19.10, 24.00)
TST (mm)	10.50 (7.00, 16.50)	13.49 (8.50, 17.00)	0.0037	9.00 (6.50, 14.00)	15.50 (10.00, 18.00)	< 0.0001	11.50 (7.50, 16.50)
SST (mm)	7.50 (5.00, 10.00)	8.50 (6.00, 13.00)	0.0048	7.50 (5.00, 10.50)	8.50 (6.50, 12.00)	0.0015	8.00 (5.50, 11.00)
AST (mm)	9.50 (7.00, 20.00)	13.50 (8.00, 22.75)	0.0230	9.00 (6.50, 14.50)	16.50 (9.00, 23.50)	< 0.0001	12.00 (7.50, 21.00)
BMI (kg/m^2^)	18.48 (16.23, 20.31)	17.79 (15.70, 20.07)	0.0631	18.12 (15.92, 19.66)	17.99 (15.84, 20.63)	0.7734	18.03 (15.90, 20.19)
WHtR	0.41 (0.40, 0.44)	0.41 (0.39, 0.44)	0.0322	0.41 (0.39, 0.44)	0.41 (0.39, 0.44)	0.3449	0.41 (0.39, 0.44)
ABSI-OR	0.0740 (0.0720, 0.0776)	0.0738 (0.0712, 0.0781)	0.6833	0.0744 (0.0724, 0.0783)	0.0735 (0.0711, 0.0771)	0.0095	0.0739 (0.0716, 0.0778)
ABSI-CN	0.1368 (0.1329, 0.1418)	0.1358 (0.1314, 0.1411)	0.1210	0.1368 (0.1330, 0.1426)	0.1354 (0.1313, 0.1408)	0.0281	0.1362 (0.1322, 0.1414)
C-index	1.11 (1.08, 1.15)	1.10 (1.06, 1.15)	0.1800	1.11 (1.08, 1.16)	1.10 (1.06, 1.14)	0.0075	1.11 (1.07, 1.15)
RFM	25.07 (18.61, 32.09)	25.33 (17.98, 30.87)	0.4602	18.44 (15.28, 22.92)	31.38 (28.63, 34.79)	< 0.0001	25.22 (18.13, 31.38)
BRI	1.89 (1.62, 2.35)	1.79 (1.46, 2.27)	0.0322	1.81 (1.53, 2.27)	1.89 (1.57, 2.30)	0.3449	1.84 (1.54, 2.28)
MaHtR	0.14 (0.13, 0.15)	0.14 (0.13, 0.15)	0.9427	0.14 (0.13, 0.15)	0.14 (0.13, 0.15)	0.0271	0.14 (0.13, 0.15)
SuST (mm)	21.50 (14.00, 33.50)	27.00 (17.25, 40.75)	0.0037	18.75 (13.50, 28.5)	32.00 (19.50, 43.50)	< 0.0001	23.50 (15.00, 37.00)
BF-S (%)	16.53 (11.08, 22.63)	20.47 (13.50, 25.05)	0.0010	14.86 (10.48, 20.62)	21.93 (15.45, 26.24)	< 0.0001	18.08 (12.09, 24.21)

WC, waist circumference; MUAC, mid-upper arm circumference; TST, triceps skinfold thickness; SST, subscapular skinfold thickness; AST, abdominal skinfold thickness; BMI, body mass index; WHtR, waist-to-height ratio; ABSI-OR, a body shape index developed by Krakauer and Krakauer (27); ABSI-CN, a body shape index developed by Xu et al. (28); C-index, conicity index; RFM, relative fat mass; BRI, body roundness index; MaHtR, mid-upper arm-to-height ratio; SuST, the sum of the skinfold thicknesses; BF-S, BF% were calculated using Slaughter's equation (43). Data are presented as the Median (Q1, Q3).

Among the 367 participants, 49.59% were Tibetan and 48.77% were female, with PS, JHS, and SHS students accounting for 38.15, 38.15, and 23.70%, respectively. Significant differences were observed in anthropometric indices including WC, TST, SST, AST, WHtR, BRI, SuST, and BF-S (%) between Tibetan and Han Chinese children and adolescents (all *p* < 0.05). Similarly, significant differences were found between males and females in most anthropometric indices (all *p* < 0.05), except for MUAC, BMI, WHtR, and BRI (Table 1). Significant differences were also observed across grade groups in all anthropometric indices (all *p* < 0.0167) except for WHtR and BRI (Supplementary Table S1).

### 3.2 Lipid levels and prevalence of dyslipidemia

As shown in Table 2, differences in blood lipid profile components were primarily observed between grade groups. Elementary school students had significantly higher TC and HDL-C levels compared to junior high school students, while junior high school students showed significantly lower LDL-C levels than high school students (all *p* < 0.0167). No significant differences were found in blood lipid profile components between ethnic groups or between males and females.

**Table 2:** Plasma lipid levels of participants stratified by ethnicity, sex, and grade group.

**Population subgroups**	**N**	**TC (mmol/L)**	**LDL-C (mmol/L)**	**HDL-C (mmol/L)**	**TG (mmol/L)**
**Ethnicity**					
Tibetan	182	3.59 (3.23, 3.98)	1.85 (1.58, 2.22)	1.26 (1.10, 1.43)	0.83 (0.65, 1.08)
Han Chinese	185	3.69 (3.28, 4.10)	1.92 (1.66, 2.32)	1.26 (1.10, 1.44)	0.86 (0.68, 1.23)
*p*		0.203	0.061	0.795	0.111
**Sex**					
Male	188	3.62 (3.25, 4.07)	1.90 (1.58, 2.29)	1.26 (1.12, 1.43)	0.83 (0.65, 1.11)
Female	179	3.60 (3.25, 4.03)	1.86 (1.65, 2.26)	1.25 (1.08, 1.45)	0.86 (0.68, 1.22)
*p*		0.904	0.783	0.419	0.132
**Grade by school level**					
PS	140	3.77 (3.35, 4.06)	1.95 (1.64, 2.27)	1.34 (1.16, 1.53)	0.88 (0.70, 1.15)
JHS	140	3.51 (3.16, 3.83)^**a**^	1.76 (1.57, 2.05)^**a**^	1.20 (1.03, 1.37)^**a**^	0.83 (0.65, 1.14)
SHS	87	3.70 (3.21, 4.14)	2.02 (1.66, 2.45)^**b**^	1.27 (1.12, 1.43)	0.82 (0.67, 1.12)
*p*		0.0046	0.0007	< 0.0001	0.647
**Total**	367	3.61 (3.25, 4.04)	1.90 (1.61, 2.26)	1.26 (1.10, 1.43)	0.85 (0.66, 1.14)

^a^Compared with PS, the differences after Bonferroni correction were statistically significant (*p* < 0.0167); ^b^Compared with JHS, the differences after Bonferroni correction were statistically significant (*p* < 0.0167); PS, primary school; JHS, junior high school; SHS, senior high school; TC, total cholesterol; LDL-C, low-density lipoprotein cholesterol; HDL-C, high-density lipoprotein cholesterol; TG, triglycerides. Data are presented as the Median (Q1, Q3).

Table 3 shows the prevalence of abnormal lipid components and overall dyslipidemia according to the ECC criteria. Among participants, 2.18% were diagnosed with high TC, 1.91% with high LDL-C, 17.98% with low HDL-C, and 6.27% with high TG. The overall prevalence of dyslipidemia was 24.25%, with significantly more females showing dyslipidemia than males. Junior high school students had the highest prevalence of dyslipidemia among the three grade groups, significantly higher than elementary school students. Han Chinese students showed a higher prevalence of dyslipidemia compared to Tibetan students; although the ethnic difference in overall prevalence was not statistically significant, Han Chinese students had a significantly higher prevalence of high TG than Tibetan students.

**Table 3:** Prevalence of dyslipidemia of participants stratified by ethnicity, sex, and grade group according to ECC criteria.

**Population subgroups**	**N**	**TC↑**	**LDL-C↑**	**HDL-C↓**	**TG↑**	**Dyslipidemia**
**Ethnicity**						
Tibetan	182	3 (0.82%)	3 (0.82%)	33 (8.99%)	6 (1.63%)	39 (10.63%)
Han Chinese	185	5 (1.36%)	4 (1.09%)	33 (8.99%)	17 (4.63%)	50 (13.62%)
*p*		0.4892	0.719	0.9415	0.0199	0.2109
**Sex**						
Male	188	2 (0.54%)	2 (0.54%)	28 (7.63%)	8 (2.18%)	37 (10.08%)
Female	179	6 (1.63%)	5 (1.36%)	38 (10.35%)	15 (4.09%)	52 (14.17%)
*p*		0.1335	0.226	0.1142	0.1032	0.0363
**Grade by school level**						
PS	140	5 (1.36%)	3 (0.82%)	13 (3.54%)	4 (1.09%)	21 (5.72%)
JHS	140	1 (0.27%)	1 (0.27%)	37 (10.08%)^a^	13 (3.54%)	44 (11.99%)^a^
SHS	87	2 (0.54%)	3 (0.82%)	16 (4.36%)	6 (1.63%)	24 (6.54%)
*p*		0.2609	0.3311	0.0009	0.082	0.0041
**Total**	367	8 (2.18%)	7 (1.91%)	66 (17.98%)	23 (6.27%)	89 (24.25%)

^a^Compared with PS, the differences after Bonferroni correction were statistically significant (*p* < 0.0167); PS, primary school; JHS, junior high school; SHS, senior high school; TC, total cholesterol; LDL-C, low-density lipoprotein cholesterol; HDL-C, high-density lipoprotein cholesterol; TG, triglycerides. Data are presented as the *N* (%).

### 3.3 Diagnostic value of anthropometric indices in identifying dyslipidemia

**Figure 1:**
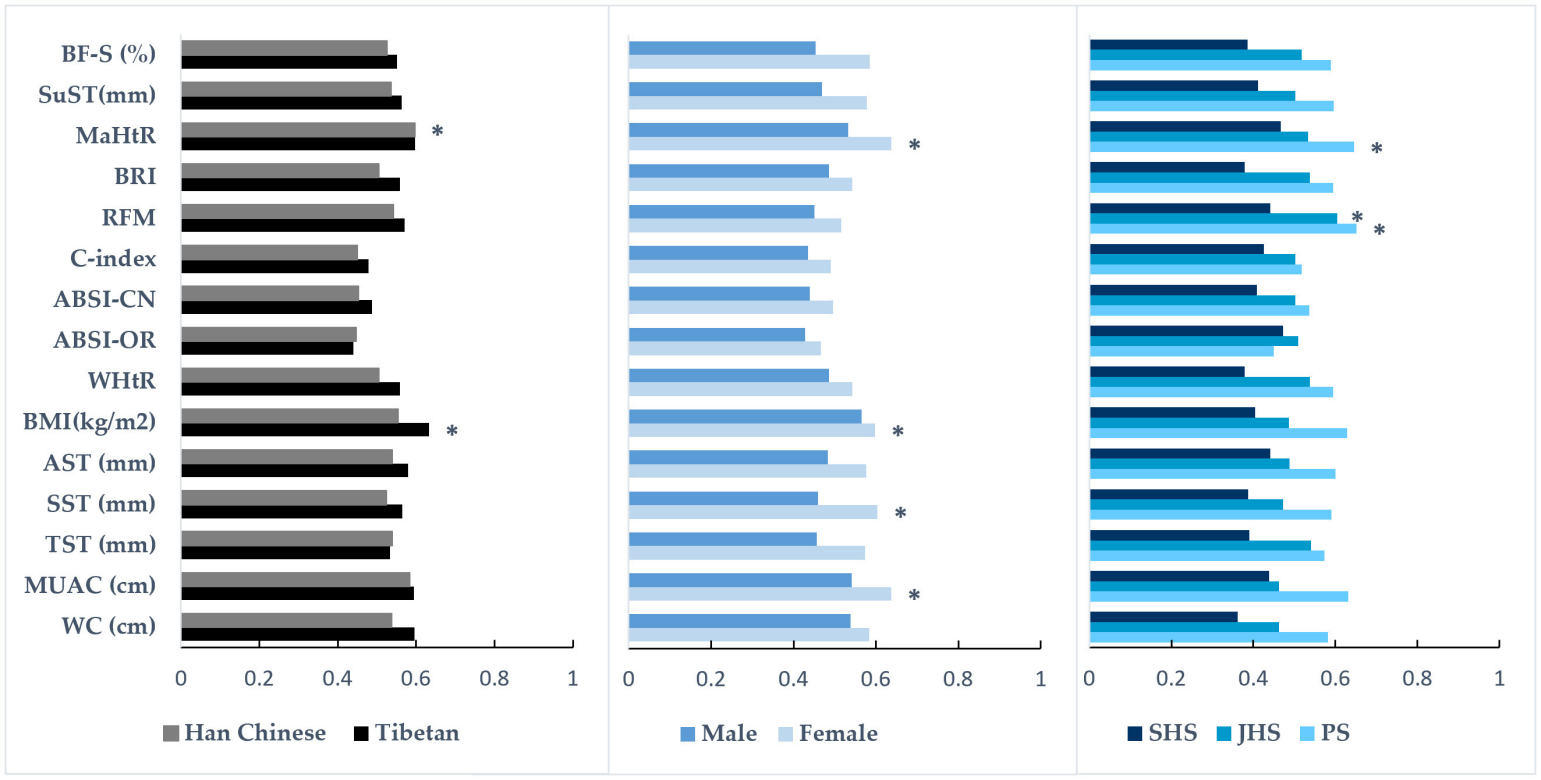
The ability of various anthropometric indices to predict the presence of dyslipidemia in population subgroups. The bar graph shows the area under the receiver operating characteristic (ROC) curve values; *the indice has diagnostic significance for dyslipidemia; PS, primary school; JHS, junior high school; SHS, senior high school; WC, waist circumference; MUAC, mid-upper arm circumference; TST, triceps skinfold thickness; SST, subscapular skinfold thickness; AST, abdominal skinfold thickness; BMI, body mass index; WHtR, waist-to-height ratio; ABSI-OR, a body shape index developed by Krakauer and Krakauer (27); ABSI-CN, a body shape index developed by Xu et al. (28); C-index, conicity index; RFM, relative fat mass; BRI, body roundness index; MaHtR, mid-upper arm-to-height ratio; SuST, the sum of the skinfold thicknesses; BF-S, BF% were calculated using Slaughter's equation (43).

Table 4 and Figure 1 show the areas under the ROC curves for different anthropometric indices in predicting dyslipidemia across the total sample and population subgroups (detailed AUC values are available in Supplementary Table S2). Overall, the majority of anthropometric indices had AUC values below 0.6 and failed to correctly identify dyslipidemia in plateau children and adolescents. Only 5 indices—MUAC, SST, BMI, RFM, and MaHtR—demonstrated significant predictive accuracy in the total sample and/or population subgroups, albeit with relatively low AUC values (0.583–0.651).

**Table 4:** Area under ROC curves (95% CI) of anthropometric indices to predict dyslipidemia.

**Anthropometric indices**	**AUC**	**(95%CI)**	**SE**	***p*-value**
WC (cm)	0.552	0.489, 0.615	0.032	0.142
MUAC (cm)	0.588	0.526, 0.650	0.032	0.013
TST (mm)	0.546	0.479, 0.614	0.034	0.188
SST (mm)	0.550	0.487, 0.614	0.033	0.153
AST (mm)	0.563	0.498, 0.628	0.033	0.075
BMI (kg/m^2^)	0.583	0.521, 0.645	0.032	0.018
WHtR	0.522	0.454, 0.590	0.035	0.530
ABSI-OR	0.440	0.375, 0.504	0.033	0.086
ABSI-CN	0.462	0.395, 0.529	0.034	0.286
C-index	0.457	0.391, 0.523	0.034	0.220
RFM	0.555	0.484, 0.625	0.036	0.120
BRI	0.522	0.454, 0.590	0.035	0.530
MaHtR	0.598	0.534, 0.663	0.033	0.005
SuST (mm)	0.558	0.493, 0.623	0.033	0.099
BF-S (%)	0.549	0.483, 0.615	0.034	0.160

AUC, area under the ROC curve; SE, Standard error; WC, waist circumference; MUAC, mid-upper arm circumference; TST, triceps skinfold thickness; SST, subscapular skinfold thickness; AST, abdominal skinfold thickness; BMI, body mass index; WHtR, waist-to-height ratio; ABSI-OR, a body shape index developed by Krakauer and Krakauer (27); ABSI-CN, a body shape index developed by Xu et al. (28); C-index, conicity index; RFM, relative fat mass; BRI, body roundness index; MaHtR, mid-upper arm-to-height ratio; SuST, the sum of the skinfold thicknesses; BF-S, BF% were calculated using Slaughter's equation (43).

Further subgroup analyses revealed that among Tibetan students, only BMI showed significant predictive accuracy, while among Han Chinese students, only MaHtR demonstrated significant predictive accuracy. In females, four indices could significantly identify dyslipidemia, ranked by AUC values as follows: BMI, SST, MUAC, and MaHtR (0.598–0.638). Among elementary school students, only RFM and MaHtR showed significant predictive accuracy, while in middle school students, only RFM demonstrated significant predictive accuracy. No indices could significantly identify dyslipidemia in males or high school students. The differences in AUC values among multiple indices with significant predictive accuracy within the same population were not statistically significant (all *p* > 0.05, Figure 1, Supplementary Table S3).

## 4 Discussion

To our knowledge, this is the first study reporting on the prevalence of dyslipidemia among children and adolescents in the Tibetan plateau region, as well as the first to describe the ability of a wide range of anthropometric indices to predict dyslipidemia in this population. The study findings not only address the knowledge gap for this unique geographic and demographic segment but also provide important evidence to guide cardiovascular health prevention and intervention strategies for children and adolescents in high-altitude areas. Our findings reveal three key patterns: high overall prevalence, ethnic disparities, and limited utility of anthropometric indices.

Our study found that the prevalence of dyslipidemia among children and adolescents in the Tibetan plateau region was higher than the national average (45–47). This result might reflect the unique influence of the high-altitude environment on lipid metabolism, leading to compensatory changes in the body's lipid metabolic patterns. High-altitude-related hypoxia has been shown to play an important role in the regulation of lipid metabolism (48), and hypoxia has been demonstrated to promote lipolysis (9), potentially increasing the levels of free fatty acids and TG in the blood, thereby causing or exacerbating dyslipidemia. Some animal studies have also indicated that hypoxia can affect adipose tissue function and lipid profiles (49, 50). Consistent with observations from other studies (36, 51, 52), low HDL-C (17.77%) was the most common lipid abnormality among children and adolescents. Similar findings have been reported in studies of high-altitude adult populations in Lhasa [3,660 m; (53)], the Peruvian highlands [4,100 m; (54)], and northern Chile [2,000–4,500 m; (55)], where low HDL-C prevalence was high. This suggests that the impact of high-altitude hypoxic environments on lipid metabolism patterns may be predominantly manifested in changes to HDL-C levels.

Noteworthy ethnic disparities were observed in the prevalence of dyslipidemia. The prevalence of dyslipidemia was higher among Han Chinese students compared to their Tibetan counterparts, especially high TG, which was contrary to our initial hypothesis. Given the characteristics of high-fat and high-cholesterol content in the traditional Tibetan diet (12), we originally expected a higher rate of dyslipidemia in Tibetan students. This unexpected finding may reflect the specific metabolic patterns that Tibetan populations have developed through long-term adaptation to the high-altitude environment, whereas the Han Chinese immigrants from low-altitude regions and their descendants may still be in the process of physiological adaptation. The large-scale economic transition from traditional to modern lifestyles in the Tibet Autonomous Region may have led to changes in dietary habits (53), ultimately affecting population-level health changes. Since the majority of students consume modernized dietary patterns in school cafeterias on school days, Tibetan school-age children and adolescents are less influenced by the traditional high-fat Tibetan dietary patterns compared to their parents' generation. Finally, differences in physical activity levels may be another possible explanation, as substantial research evidence has shown that the adjustment of physical activity can have a positive impact on the blood lipid profile (56, 57). Our previous study found that Tibetan students had significantly higher physical activity levels than Han Chinese students (6). The difference in the prevalence of dyslipidemia between Tibetan and Han Chinese students underscores the importance of considering ethnic-specific factors in high-altitude epidemiological research.

As previously reported (5, 47), the prevalence of dyslipidemia was significantly higher in girls than in boys. This sexual disparity may be attributed to several factors. Physiologically, females have higher skinfold thickness and BF% compared to males (see Table 1), and excessive accumulation of adipose tissue promotes adiposopathy, leading to abnormal circulating lipid levels (58). Behaviorally, females engage less in moderate-to-vigorous physical activity (6), which is a risk factor for lipid metabolism disorders (59). These findings highlight the necessity of fully considering sexual specificity when formulating intervention strategies for dyslipidemia in children and adolescents living in highland areas.

Our study revealed an apparently paradoxical yet intriguing phenomenon: junior high school students exhibited significantly lower levels of TC, LDL-C, and HDL-C compared to primary school students, while simultaneously showing the highest prevalence of dyslipidemia. This finding warrants a multifaceted interpretation. During puberty, hormonal changes associated with pubertal growth spurt and progressive maturation lead to increased cholesterol demands, resulting in decreased blood lipid levels (60). In our study, the lower TC and LDL-C levels observed in junior high school students corroborated this physiological mechanism, suggesting that children and adolescents in the Tibetan Plateau share certain physiological changes with their peers in other regions. However, since HDL-C is considered “good” cholesterol, and its reduction increases the risk of dyslipidemia and cardiovascular disease [CVD; (61)], the predominance of low HDL-C among junior high school students explains the concurrent observation of lower TC and LDL-C levels yet higher dyslipidemia prevalence. This phenomenon potentially reveals the complexity and uniqueness of age-related lipid profile characteristics among plateau children and adolescents. Larger population-based longitudinal studies are needed to better understand the developmental trajectory of lipid metabolism in plateau environments.

Since dyslipidemia in adolescence increases the risk of cardiovascular disease throughout the life course (5), and lipid levels are reversible, screening and controlling dyslipidemia from childhood is a practical health strategy for preventing the risk of adult cardiovascular disease mortality (20). Rapid, safe, inexpensive, and non-invasive screening for dyslipidemia in the pediatric population is a prerequisite for achieving this goal. It is generally believed that excessive body fat is closely related to blood lipids (19); therefore, anthropometric indicators for assessing body shape or fat distribution have been used to explore their applicability in assessing lipid levels. Previous studies have reported that most of these anthropometric indicators can well predict dyslipidemia in adults at low altitudes (17, 35, 62). However, children and adolescents are in a stage of rapid changes in physical development, and the anthropometric indicators used to assess lipid levels may differ from those in adults, and research in this area is still limited, especially in highland areas. Therefore, we systematically evaluated the predictive ability of 16 anthropometric indices for dyslipidemia in children and adolescents living in high-altitude areas. Overall, the predictive ability of these indices was relatively weak, with the AUC of most indices failing to reach 0.6. Only five indices—BMI, SST, MUAC, MaHtR, and RFM—showed weak yet promising diagnostic value.

As a widely used tool for assessing obesity, BMI reflects the overall distribution of body fat (63). Previous studies have reported that BMI performs well in identifying dyslipidemia in children and adolescents at low altitudes (22, 64). In the present study, although BMI had a lower predictive value, it was one of the few indicators that could significantly correctly classify dyslipidemia. This may be related to the fact that the main phenotypes of dyslipidemia in this study were low HDL-C and high TG. A systematic review (65) indicated that BMI is a powerful indicator for predicting abnormalities in TG and HDL-C rather than TC and LDL-C.

Skinfold thickness is considered the gold standard for describing body fat distribution (66) and can assess body obesity more directly and accurately than BMI (67). However, among the five skinfold thickness-related indicators in this study, only SST had predictive value for dyslipidemia in Tibetan highland girls (AUC = 0.604, *p* < 0.05). This result suggests that long-term exposure to a hypoxic environment may lead to changes in fat distribution patterns, making it difficult for traditional skinfold thickness indicators to accurately reflect the risk of dyslipidemia. Moreover, the reliability of skinfold thickness measurements has been controversial (24), as factors such as the grip strength of the tester, the pressure applied by the caliper, and the age, sex, and skin temperature of the subject can all affect the measurement results (68), which may limit the predictive ability of skinfold thickness and its derived indicators. Finally, the low predictive efficacy of BF-S may be related to the fact that the Slaughter skinfold equation was established based on data from children at low altitudes, and the assumed “subcutaneous fat-to-total fat ratio” in the Slaughter equation may not apply to highland populations.

MUAC and MaHtR are two relatively new anthropometric indicators, with the latter being an index of the former standardized by height. Both have been shown to be reliable tools for detecting overweight and obesity in school-age children (69, 70). However, few studies have investigated the association between MUAC/MaHtR and lipid levels in children and adolescents, with only two articles reporting their significant association with dyslipidemia in school-age children (20, 34). Similar to these studies, our study demonstrated that both MUAC and MaHtR have the ability to correctly predict dyslipidemia in highland children and adolescents. RFM is also a new anthropometric index that has been found to estimate an individual's body fat percentage more accurately than BMI (29, 30). Due to its recent development, there are relatively few studies exploring its association with dyslipidemia. Limited studies have shown that RFM has a high predictive ability for dyslipidemia in adults (35, 71). Our study, is the first to confirm the diagnostic efficacy of RFM for dyslipidemia in children and adolescents. Although the AUC values of these indicators are not high, these findings are essential for further understanding the potential application value of MUAC, MaHtR, and RFM as simple anthropometric indicators in preventing metabolic diseases in children and adolescents, and also serve as a valuable addition to the lack of evidence in this field.

An interesting finding is that anthropometric indicators have greater predictive value in populations with lower educational levels (primary and junior high school students). In contrast to the high school group, where no anthropometric indicators were found to effectively predict dyslipidemia, RFM and MaHtR showed some predictive ability in primary and junior high school groups. Moreover, the AUC of RFM for predicting dyslipidemia was higher in primary school students than in junior high school students (AUC: 0.651 vs. 0.604). This result is in direct opposition to the conclusion of Quadros et al.'s study on Brazilian children and adolescents (36), which observed that regardless of sex, no anthropometric indicators could significantly predict dyslipidemia in the 8–9 age group, while the 16–18 age group had the highest predictive accuracy. The reasons for this discrepancy may involve multiple factors.

First, the sample size of the high school group was significantly lower than that of the primary and junior high school groups, which may have affected the power of statistical tests, making it difficult to detect potential associations between anthropometric indicators and dyslipidemia in the high school population. Second, although changes in the lipid profile may be more influenced by hormonal fluctuations during puberty than by the accumulation of body fat (72), children exposed to a hypoxic environment above 3,000 meters for an extended period exhibit unique growth patterns (73) and a higher risk of developmental delay (74). These characteristics may lead to a delayed onset of puberty in primary school students in the Tibetan region, thereby prolonging the “fat-dominant period” and maintaining the correlation between anthropometric indicators and dyslipidemia. In contrast, although high school students have an older chronological age, their physiological maturity may be closer to that of junior high students in plain areas. Therefore, the complex influence of pubertal hormonal changes on lipid metabolism may mask the predictive role of single anthropometric indicators. Furthermore, the multidimensional interaction between genetics, environment, and lifestyle factors such as diet and physical activity may reshape the age trajectory of the body fat-blood lipid relationship in children and adolescents in highland areas. This complex interaction effect leads to different performances of anthropometric indicators in predicting dyslipidemia among children and adolescents in highland areas compared to those in plain areas. Future studies should adopt a longitudinal design to deeply analyze the temporal relationship between hormones, body fat, and blood lipids in children and adolescents in the Tibetan highland region, and to clarify the key time points for using anthropometric indicators to screen for metabolic risk factors such as dyslipidemia. This will provide important scientific evidence for developing personalized health management and early intervention strategies suitable for high-altitude areas.

This study also has several limitations that must be considered. First, the cross-sectional nature of the study precludes the establishment of any causal relationships based on the study data. Second, the participants were all from elementary and secondary schools in the Chengguan District of Lhasa City, without including children and adolescents from other altitude regions, which may limit the generalizability of the research results to the entire high-altitude area. However, it is important to emphasize that the Chengguan District is home to more than half of the senior high schools and two-thirds of the junior high schools in the Tibet Autonomous Region, and sampling in this region is most feasible, so the obtained sample still has a certain degree of representativeness. Moreover, our study lacks information on lifestyle factors and family history of dyslipidemia, which may also influence lipid levels and potentially distort our findings to some extent. Finally, the sample size of the senior high school group was significantly smaller than the elementary and junior high school groups, which may have affected the research results.

## 5 Conclusions

This study demonstrated a high prevalence of dyslipidemia among children and adolescents aged 8 to 17 years on the Tibetan Plateau. Han Chinese students exhibited higher dyslipidemia prevalence compared to their Tibetan counterparts, with higher rates observed among females than males. Junior high school students demonstrated the highest prevalence among the three grade groups. Low HDL-C emerged as the predominant dyslipidemia phenotype. Most anthropometric indices showed limited capability in diagnosing dyslipidemia in high-altitude children and adolescents, although five indices—BMI, SST, MUAC, MaHtR, and RFM—demonstrated promising potential. However, given their relatively low diagnostic value and applicability to a limited population, the actual utility of these indices as screening tools for dyslipidemia warrants further investigation. This finding suggests that a single indicator or existing anthropometric indicators may not be sufficient to fully meet the screening needs for dyslipidemia in children and adolescents living in high-altitude areas. Therefore, future research should further explore the optimal combination of indicators or develop new anthropometric indicators to more accurately reflect the body fat distribution characteristics and their relationship with dyslipidemia in this specific population.

## Data Availability

The raw data supporting the conclusions of this article will be made available by the authors, without undue reservation.
