# Supplementary material for: Prevalence of dyslipidemia and predictive value of anthropometric indicators among children and adolescents in the Tibetan Plateau
Source: Front Nutr. 2025 Mar 28;12:1531197. doi: 10.3389/fnut.2025.1531197 (PMC11985435; doi:10.3389/fnut.2025.1531197)
Supplement: Supplementary file 1 [file Table_1.docx]

Supplementary Material

**Table S1 Demographic characteristics of participants in different grade groups**

| **Variables** | **PS students** | **JHS students** | **SHS students** |
| --- | --- | --- | --- |
| N | 140(38.15%) | 140(38.15%) | 87(23.70%) |
| Age (years) | 10.91(10.30,11.70)**^b^** | 13.9(13.00,14.80)**^c^** | 16.90(16.30,17.60)**^a^** |
| Height (cm) | 140.85(134.43,145.75)**^b^** | 158.20(152.47,163.92)**^c^** | 164.40(159.19,170.00)**^a^** |
| Weight (kg) | 31.05(27.53,35.33)**^b^** | 48.70(44.03,53.58)**^c^** | 53.30(49.20,62.50)**^a^** |
| WC (cm) | 57.00(54.00,61.75)**^b^** | 66.50(62.25,70.07)**^c^** | 68.90(64.50,73.00)**^a^** |
| MUAC (cm) | 18.50(17.30,20.50)**^b^** | 23.00(21.00,24.15)**^c^** | 24.00(22.50,25.30)**^a^** |
| TST (mm) | 8.50(6.50,13.00)**^b^** | 13.75(8.13,18.00) | 15.50(10.00,19.00)**^a^** |
| SST (mm) | 5.00(4.13,7.50)**^b^** | 9.00(7.00,12.50)**^c^** | 10.50(8.00,14.00)**^a^** |
| AST (mm) | 7.75(5.00,11.88)**^b^** | 16.25(9.00,24.50) | 19.50(9.50,27.00)**^a^** |
| BMI(kg/m^2^) | 15.75(14.56,17.11)**^b^** | 19.10(17.73,20.72)**^c^** | 20.06(18.38,22.39)**^a^** |
| WHtR | 0.41(0.39,0.43) | 0.41(0.39,0.44) | 0.42(0.39,0.45) |
| ABSI-OR | 0.0776(0.0740,0.0806)**^b^** | 0.0733(0.0712,0.0753) | 0.0725(0.0702,0.0740)**^a^** |
| ABSI-CN | 0.1385(0.1332,0.1432)**^b^** | 0.1359(0.1316,0.1400) | 0.1343(0.1314,0.1401)**^a^** |
| C-index | 1.13(1.08,1.16)**^b^** | 1.10(1.07,1.13) | 1.09(1.06,1.13)**^a^** |
| RFM | 26.36(19.95,32.31) | 26.67(18.79,32.53)**^c^** | 21.07(15.10,28.26)**^a^** |
| BRI | 1.78(1.50,2.20) | 1.88(1.57,2.32) | 1.95(1.53,2.39) |
| MaHtR | 0.13(0.12,0.14)**^b^** | 0.14(0.13,0.15) | 0.14(0.14,0.16)**^a^** |
| SuST(mm) | 17.00(12.13,23.00)**^b^** | 31.50(18.13,43.38) | 34.00(21.50,47.00)**^a^** |
| BF-S (%) | 13.43(9.97,18.08)**^b^** | 21.51(14.86,25.97) | 22.63(17.33,26.78)**^a^** |
| Notes: a, Compared with PS, the differences after Bonferroni correction were statistically significant (p < 0.0167); b, Compared with JHS, the differences after Bonferroni correction were statistically significant (p < 0.0167); c, Compared with SHS, the differences after Bonferroni correction were statistically significant (p < 0.0167); PS—primary school, JHS—junior high school, SHS—senior high school, WC—waist circumference, MUAC—mid-upper arm circumference, TST—triceps skinfold thickness, SST—subscapular skinfold thickness, AST—abdominal skinfold thickness, BMI—body mass index, WHtR—waist-to-height ratio, ABSI-OR—a body shape index developed by Krakauer et al. (1), ABSI-CN—a body shape index developed by Xu et al. (2), C-index—conicity index, RFM—relative fat mass, BRI—body roundness index, MaHtR—mid-upper arm-to-height ratio, SuST—the sum of the skinfold thicknesses, BF-S—BF% were calculated using Slaughter’s equation(3). Data are presented as the Median(Q1, Q3). | | | |

**Table S2 Area under ROC curves (95% CI) for anthropometric indices in predicting dyslipidemia across population subgroups (95% CI).**

| **Anthropometric**  **indices** | **Ethnicity** | |  | **Gender** | |  | **Grade by School Level** | | |
| --- | --- | --- | --- | --- | --- | --- | --- | --- | --- |
|  | **Tibetan** | **Han Chinese** |  | **Male** | **Female** |  | **PS** | **JHS** | **SHS** |
| WC (cm) | 0.596(0.505,0.687) | 0.537(0.449,0.625) |  | 0.538(0.444,0.633) | 0.583(0.497,0.669) |  | 0.582(0.441,0.723) | 0.462(0.362,0.562) | 0.362(0.244,0.480) |
| MUAC (cm) | 0.594(0.504,0.684) | 0.583(0.498,0.669) |  | 0.541(0.449,0.633) | 0.637(0.553,0.722)* |  | 0.632(0.495,0.769) | 0.462(0.363,0.561) | 0.439(0.318,0.560) |
| TST (mm) | 0.534(0.427,0.640) | 0.538(0.449,0.627) |  | 0.456(0.357,0.555) | 0.574(0.488,0.660) |  | 0.573(0.437,0.708) | 0.541(0.438,0.644) | 0.390(0.265,0.515) |
| SST (mm) | 0.564(0.467,0.661) | 0.524(0.438,0.610) |  | 0.459(0.366,0.551) | 0.604(0.519,0.688)* |  | 0.590(0.461,0.720) | 0.473(0.371,0.575) | 0.388(0.262,0.513) |
| AST (mm) | 0.579(0.484,0.673) | 0.539(0.449,0.630) |  | 0.484(0.385,0.583) | 0.576(0.49,0.661) |  | 0.600(0.463,0.737) | 0.488(0.386,0.589) | 0.441(0.311,0.572) |
| BMI(kg/m2) | 0.633(0.544,0.723)* | 0.553(0.468,0.639) |  | 0.565(0.469,0.660) | 0.598(0.513,0.682)* |  | 0.628(0.493,0.764) | 0.487(0.387,0.588) | 0.405(0.281,0.529) |
| WHtR | 0.559(0.458,0.660) | 0.504(0.413,0.595) |  | 0.486(0.384,0.589) | 0.543(0.452,0.635) |  | 0.594(0.442,0.746) | 0.538(0.436,0.640) | 0.379(0.263,0.495) |
| ABSI-OR | 0.440(0.347,0.533) | 0.446(0.356,0.536) |  | 0.428(0.330,0.527) | 0.467(0.379,0.556) |  | 0.450(0.331,0.569) | 0.509(0.406,0.613) | 0.473(0.345,0.601) |
| ABSI-CN | 0.488(0.391,0.586) | 0.453(0.361,0.545) |  | 0.439(0.337,0.540) | 0.496(0.405,0.586) |  | 0.536(0.405,0.667) | 0.502(0.397,0.607) | 0.409(0.288,0.530) |
| C-index | 0.479(0.383,0.574) | 0.450(0.359,0.542) |  | 0.435(0.334,0.536) | 0.491(0.401,0.581) |  | 0.518(0.393,0.643) | 0.502(0.397,0.607) | 0.425(0.302,0.547) |
| RFM | 0.571(0.465,0.678) | 0.542(0.447,0.637) |  | 0.451(0.342,0.560) | 0.516(0.419,0.613) |  | 0.651(0.511,0.791)* | 0.604(0.506,0.702)* | 0.441(0.312,0.570) |
| BRI | 0.559(0.458,0.660) | 0.504(0.413,0.595) |  | 0.486(0.384,0.589) | 0.543(0.452,0.635) |  | 0.594(0.442,0.746) | 0.538(0.436,0.640) | 0.379(0.263,0.495) |
| MaHtR | 0.597(0.500,0.693) | 0.596(0.509,0.684)* |  | 0.533(0.437,0.630) | 0.638(0.552,0.724)* |  | 0.645(0.501,0.790)* | 0.534(0.433,0.634) | 0.467(0.341,0.592) |
| SuST(mm) | 0.563(0.465,0.662) | 0.536(0.447,0.625) |  | 0.469(0.372,0.566) | 0.578(0.493,0.662) |  | 0.596(0.462,0.730) | 0.502(0.402,0.603) | 0.411(0.284,0.538) |
| BF-S (%) | 0.551(0.448,0.653) | 0.526(0.438,0.614) |  | 0.454(0.360,0.549) | 0.585(0.499,0.671) |  | 0.589(0.459,0.718) | 0.518(0.416,0.621) | 0.386(0.261,0.512) |
| Notes: *, the indice has diagnostic significance for dyslipidemia; PS—primary school, JHS—junior high school, SHS—senior high school; WC—waist circumference, MUAC—mid-upper arm circumference, TST—triceps skinfold thickness, SST—subscapular skinfold thickness, AST—abdominal skinfold thickness, BMI—body mass index, WHtR—waist-to-height ratio, ABSI-OR—a body shape index developed by Krakauer et al. (1), ABSI-CN—a body shape index developed by Xu et al. (2), C-index—conicity index, RFM—relative fat mass, BRI—body roundness index, MaHtR—mid-upper arm-to-height ratio, SuST—the sum of the skinfold thicknesses, BF-S—BF% were calculated using Slaughter’s equation(3). | | | | | | | | | |

**Table S3 Performance differences between ROC curves compared using the delong test.**

| **Variables to compare** | **Z** | ***p* value** | **AUC Difference** | **SE Difference** | **(95%CI)** |
| --- | --- | --- | --- | --- | --- |
| **Female** |  |  |  |  |  |
| MUAC vs. SST | 1.027 | 0.305 | 0.034 | 0.289 | -0.031, 0.098 |
| MUAC vs. BMI | 1.963 | 0.050 | 0.040 | 0.288 | 0.00005576, 0.079 |
| MUAC vs. MaHtR | -0.020 | 0.984 | -0.001 | 0.290 | -0.052, 0.051 |
| SST vs. BMI | 0.172 | 0.863 | 0.006 | 0.289 | -0.061, 0.073 |
| SST vs. MaHtR | -0.855 | 0.393 | -0.034 | 0.292 | -0.113, 0.044 |
| BMI vs. MaHtR | -1.317 | 0.188 | -0.040 | 0.291 | -0.100, 0.020 |
| **PS** |  |  |  |  |  |
| MaHtR vs. RFM | -0.081 | 0.935 | -0.006 | 0.376 | -0.140, 0.129 |
| **Total** |  |  |  |  |  |
| MaHtR vs. MUAC | 0.493 | 0.622 | 0.011 | 0.251 | -0.032, 0.054 |
| MaHtR vs. BMI | 0.675 | 0.500 | 0.015 | 0.252 | -0.03, 0.061 |
| MUAC vs. BMI | 0.297 | 0.767 | 0.005 | 0.249 | -0.026, 0.036 |
| Notes: PS—primary school, AUC—area under the ROC curve, SE—Standard error, MUAC—mid-upper arm circumference, SST—subscapular skinfold thickness, BMI—body mass index, RFM—relative fat mass, MaHtR—mid-upper arm-to-height ratio. | | | | | |

**References**

1. Krakauer NY, Krakauer JC. A New Body Shape Index Predicts Mortality Hazard Independently of Body Mass Index. *PLoS One* (2012) 7(7):e39504. Epub 20120718. doi: 10.1371/journal.pone.0039504.

2. Xu Y, Yan W, Cheung YB. Body Shape Indices and Cardiometabolic Risk in Adolescents. *Ann Hum Biol* (2015) 42(1):70-5. Epub 20140415. doi: 10.3109/03014460.2014.903998.

3. Slaughter MH, Lohman TG, Boileau RA, Horswill CA, Stillman RJ, Van Loan MD, Bemben DA. Skinfold Equations for Estimation of Body Fatness in Children and Youth. *Hum Biol* (1988) 60(5):709-23.
